# Empyema necessitans caused by methicillin-resistant *Staphylococcus aureus:* a case report and literature review

**DOI:** 10.1186/s12879-024-09062-0

**Published:** 2024-02-01

**Authors:** Tomoaki Nakamura, Kazuhiro Ishikawa, Naoki Murata, Kuniko Sato, Atsushi Kitamura, Nobuyoshi Mori, Torahiko Jinta

**Affiliations:** 1 Department of Pulmonary Medicine, Thoracic Center, St. Luke’s International Hospital, Tokyo, Japan; 2 Department of Infectious Diseases, St. Luke’s International Hospital, Tokyo, Japan; 3 St. Luke’s International University Library, Tokyo, Japan

**Keywords:** Empyema necessitans, Methicillin-resistant *Staphylococcus aureus*, Literature review, *Staphylococcus aureus* bacteremia

## Abstract

**Background:**

Empyema necessitans (EN) is a rare condition characterized by pleural infection with pus spreading into adjacent soft tissues. Although *Mycobacterium tuberculosis* and *Actinomyces israelii* are common causative agents, methicillin-resistant *Staphylococcus aureus* (MRSA) is relatively rare, but it is associated with high mortality in empyema cases. We aimed to report a unique case of EN caused by MRSA and present a literature review to better understand this rare condition.

**Case presentation:**

A 69-year-old man with a history of right ureteral stone presented with fever and left anterior thoracic pain. A physical examination revealed redness and swelling in the left thoracic region. Imaging studies confirmed EN with fluid accumulation around the sternocostal joint of the left first rib. MRSA was identified from blood and pleural fluid cultures. The patient received antimicrobial therapy, and a chest tube was inserted for drainage. Despite initial improvement, vertebral osteomyelitis was diagnosed on day 17. The antimicrobials were subsequently terminated after 6 weeks, but vertebral osteomyelitis recurred, and treatment was resumed and completed on day 215.

**Conclusion:**

EN caused by MRSA is rare, and the literature review revealed 14 cases from human sources. Positive blood cultures were observed in 40% of cases, and metastatic infections were present in 30% of cases. Osteomyelitis was the most common type of metastatic lesion. All the patients underwent drainage. Patients with MRSA-associated EN frequently develop disseminated lesions and should therefore be carefully examined. Moreover, appropriate treatment with antibiotics and drainage is necessary for a good prognosis. Although the prognosis appeared to be favorable in our review, publication bias and treatment challenges for metastatic infections should be considered.

**Supplementary Information:**

The online version contains supplementary material available at 10.1186/s12879-024-09062-0.

## Background

Empyema necessitans (EN) refers to an infection of the pleura and the associated spread of pus beyond the pleural cavity into adjacent soft tissue structures [1]. *Mycobacterium tuberculosis* (*M. tuberculosis)* and *Actinomyces israelii* (*A. israelii)* are the most common causative organisms of EN [2–4]. In recent years, the incidence of EN has decreased with the use of antibiotics [5].

Methicillin-resistant *Staphylococcus aureus* (MRSA) is a relatively rare causative agent of empyema. Furthermore, those associated with bacteremia have a mortality rate as high as 42.1% [6]. Based on the above, EN caused by MRSA has rarely been reported, but it is expected to be more severe. We aimed to report the case of EN caused by MRSA, and owing to its rarity, we performed a literature review to investigate its complications, management, and prognosis.

## Case presentation

**Fig. 1:**
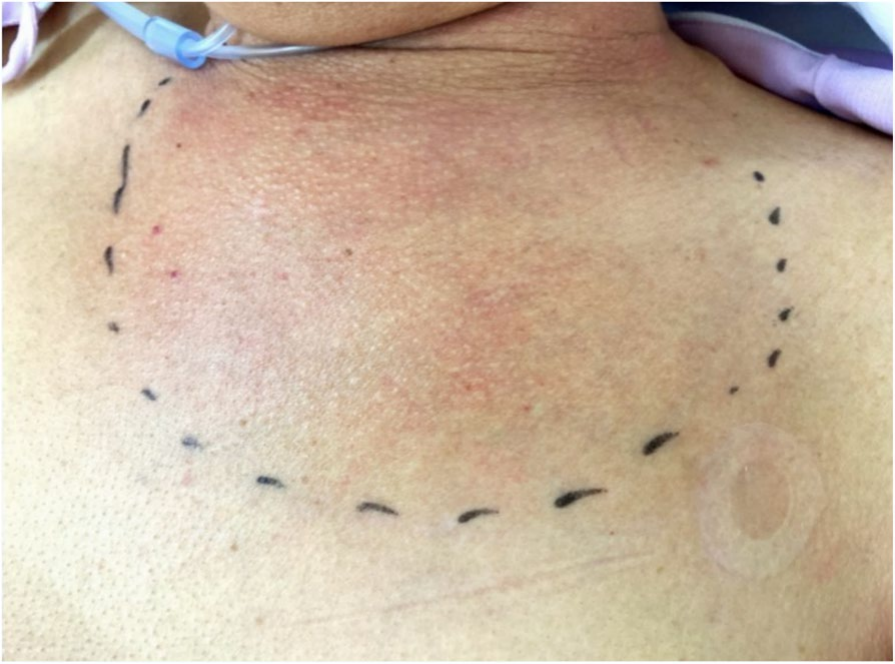
Physical examination on the day of admission. Physical examination reveals redness, heat, tenderness, and swelling in the left anterior thoracic region

A 69-year-old man with an asymptomatic right ureteral stone presented to the hospital with a chief complaint of fever that had begun one week earlier. He took no oral medications, had a 100-pack-year smoking history, and consumed 350 mL/day of beer. He had no allergies or significant family history. He had worked for many years in the tuna brokering business but had retired several months earlier and was currently unemployed. Six days before his visit, the patient developed redness and pain in the left anterior thoracic region and had difficulty raising his left arm. The day before the visit, he experienced gross hematuria and was prescribed sitafloxacin at a nearby clinic for a suspected urinary tract infection. On admission, the patient was conscious, with a Glasgow Coma Scale score of E4V5M6, temperature of 38.1°C, blood pressure of 140/80 mmHg, pulse of 99/min, respiratory rate of 28/min, and oxygen saturation of 99% (nasal cannula, 1 L/min). Physical examination revealed redness, hot tenderness, fluctuant swelling, and bulging in the left anterior thoracic region (Fig. 1). Peripheral signs suggestive of infective endocarditis were observed. No crackles were heard on auscultation, and there was no spinous process tenderness. Laboratory findings revealed the following: white blood cell count, 22,700/μL (neutrophils, 90.5%; lymphocytes, 5.5%; monocytes, 3.0%) (normal range: 3,300–8,600/L); creatinine, 0.85 mg/dL (normal range: 0.65–1.07 mg/dL); total protein, 6.9 g/dL (normal range: 6.6–8.1 g/dL); lactate dehydrogenase (LDH), 269 U/L (normal range: 124–222 U/L); glucose, 162 mg/dL (normal range: 73–109 mg/dL), and C-reactive protein, 37.8 mg/dL (normal range: 0.00–0.14 mg/dL). Urinalysis revealed occult blood 2 + and leukocytes 1 + .

**Fig. 2:**
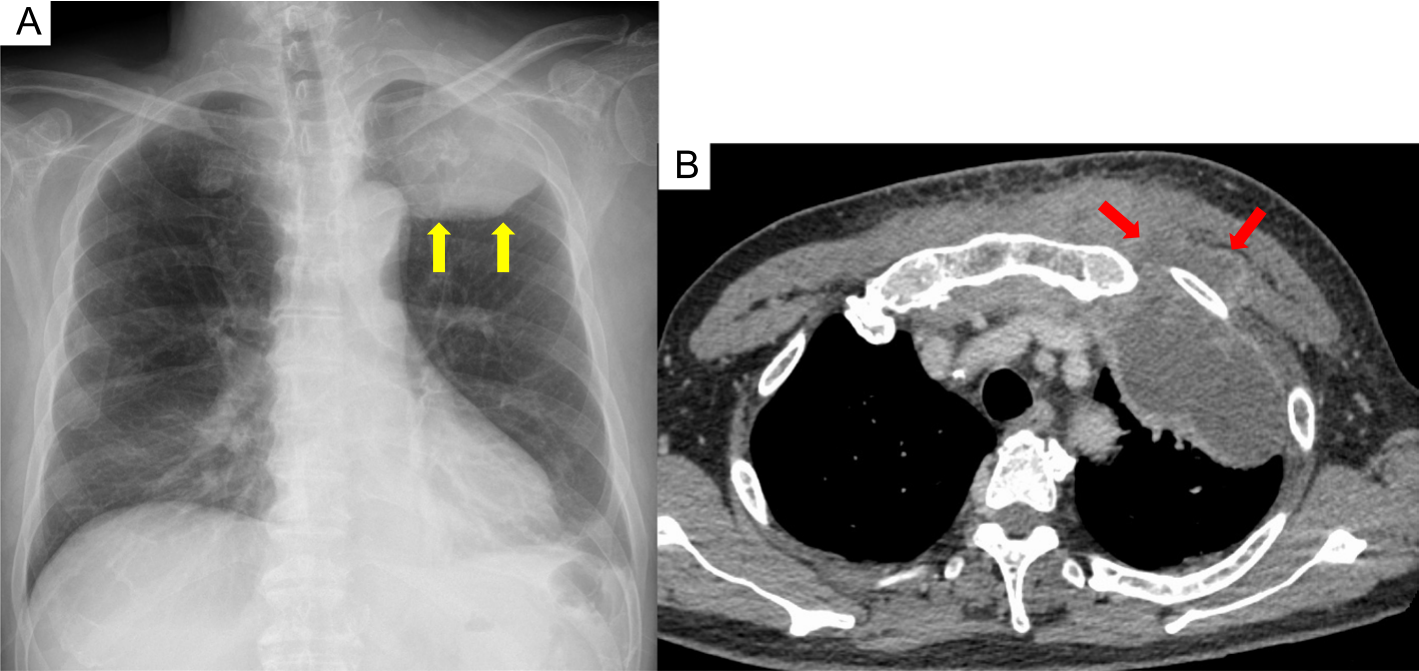
Chest radiography and contrast-enhanced chest computed tomography scan acquired on admission. **a** Chest radiography reveals an infiltrative shadow in the left upper lung field. **b** Contrast-enhanced computed tomography reveals fluid accumulation with marginal contrast enhancement around the sternocostal joint of the left first rib, extending subcutaneously

Chest radiography revealed an infiltrative shadow in the left upper lung field, and contrast-enhanced computed tomography (CT) showed fluid accumulation with marginal contrast enhancement around the sternocostal joint of the left first rib, extending subcutaneously (Fig. 2a, b). This image findings were consistent with EN. A small-bore chest tube was inserted at the same site, and purulent turbid drainage was obtained. Pleural fluid revealed a pH of 6.9, total protein of 3.9 g/dL, LDH of 3,561 U/L, glucose of 25 mg/dL, adenosine deaminase of 87.1 U/L, and total cell count of 24,900/μL (neutrophils, 98%; monocytes, 2.0%). On the same day, ampicillin/sulbactam 3 g every 6 h was started; on the second day, vancomycin (VAN) 1.25 g every 12 h was added because Gram-positive cocci in clusters were observed in the Gram stain from the blood and pleural fluid collected on admission. Acid-fast bacilli smear, culture, and polymerase chain reaction of the pleural fluid specimen were all negative. The serum trough concentration of VAN was 15–20 mg/L. On the third day, a chest radiography revealed that the infiltrative shadow in the left upper lung field was reduced; however, an infiltrative shadow in the left lower lung field appeared, and a drain was added at the site. On the fourth day, the final culture revealed MRSA in the blood and pleural fluid at the time of admission. This was confirmed using matrix-assisted laser desorption/ionization time-of-flight mass spectrometry (Bruker Biotyper, Bruker Daltonik GmbH, Bremen, Germany). The susceptibility test was performed using the MicroScan Walkaway Plus automatic system (Beckman Coulter, USA) (Table 1). A blood culture obtained on day 6 also showed persistent positivity; therefore, daptomycin 700 mg (9 mg/kg) was added every 24 h. Blood cultures obtained on day eight yielded negative results. Transthoracic echocardiography was performed twice, with one week interval, with no findings suggestive of infective endocarditis. On day 10, drainage from the chest tube was decreased, and the shadows on the chest radiograph improved; therefore, the chest tube was removed. Thereafter, the fever resolved; however, on the 17th day, the patient had fever with neck pain, and contrast-enhanced magnetic resonance imaging (MRI) revealed contrast enhancement of the vertebral body and perivertebral space at C7–T1, which led to the diagnosis of vertebral osteomyelitis. No epidural abscess was observed. The patient clinically improved and was discharged from the hospital on the 28th day because the fever gradually resolved, cervical pain tended to improve, and the antimicrobial agent was changed to oral linezolid 600 mg every 12 h.

**Table 1:** Antimicrobial susceptibility of the MRSA isolated from blood and pleural fluid in this case

Antimicrobials	MIC (μg/mL)	Susceptibility
Cefazolin	≤ 8	Resistant
Vancomycin	1	Susceptible
Gentamicin	≤ 2	Susceptible
Minomycin	≤ 2	Susceptible
Erythromycin	> 4	Resistant
Clindamycin	≤ 0.5	Susceptible
Penicillin G	> 8	Resistant
Oxacillin	> 2	Resistant
Sulfamethoxazole/trimethoprim	≤ 1	Susceptible
Ampicillin/sulbactam	≤ 8	Resistant
Levofloxacin	4	Resistant
Linezolid	≤ 1	Susceptible
Daptomycin	≤ 0.25	Susceptible
Imipenem/cilastatin	≤ 1	Susceptible

Susceptibility test is performed based on the Clinical and Laboratory Standards Institute guidelines (M100Ed33)

*Abbreviation*s: *MIC* minimal inhibitory concentration, *MRSA* methicillin-resistant *Staphylococcus aureus*

Taste disturbance due to linezolid was observed; however, chest radiography revealed a decrease in pleural effusion, and the treatment was terminated on day 58. In retrospect, the erythrocyte sedimentation rate (ESR) at this time was 80 mm/h. On day 67, the patient again presented with neck pain and fever, and contrast-enhanced CT revealed enhanced soft tissue shadows around the C7–T1 vertebral body. He was readmitted with a diagnosis of a flare-up of vertebral osteomyelitis. Therefore, we restated VAN. There was no worsening of pleural effusion on chest radiography. The patient continued VAN for 14 days and was then switched to oral sulfamethoxazole–trimethoprim (SXT) 160 mg/800 mg every 12 h. On day 125, due to elevated liver enzyme levels, the patient was administered daptomycin for three days. The enzyme levels quickly normalized and were elevated only once during this period. Subsequently, the treatment was switched to oral minocycline 100 mg every 12 h. After confirming that the ESR had normalized, treatment was terminated on day 215. No relapse has occurred since then.

## Methods of literature review

**Fig. 3:**
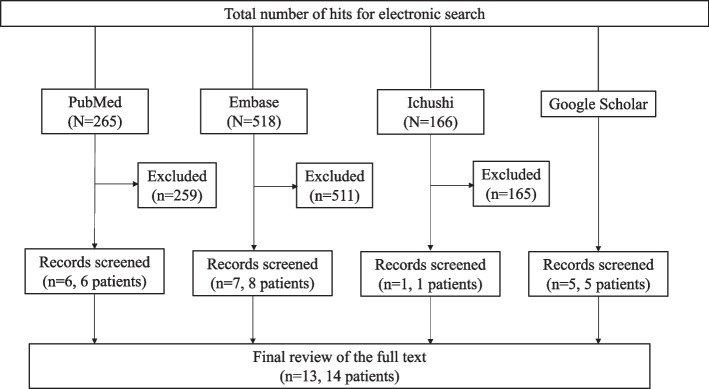
Literature review flowchart

Two authors independently reviewed the titles and abstracts of the database records, retrieved the full texts for eligibility assessment, and extracted data from the case reports. We searched for case reports of empyema due to MRSA and reviewed the images individually to determine if they qualified for EN. We searched the PubMed and Embase databases using specific keywords (Additional file 1). The following filters were applied: English or Japanese language and articles registered in the literature database until April 30, 2023. Conference abstracts were excluded. The PubMed and Embase searches generated 265 and 518 articles, respectively. Of these, 259 and 511 reports from PubMed and Embase, respectively, were excluded since either they were not case reports or case reports that did not focused on EN based on the images included. Considering that there were several reports in Japanese papers, we included only those published in Japanese to further understand the clinical characteristics of the disease by presenting more confirmed cases. To search for articles in Japanese, we used Ichushi-Web, a major Japanese database, using some keywords (Additional file 1). We examined the eligibility and the work conducted in PubMed and Embase, and finally, one case was included. We searched Google Scholar and identified five additional human cases. Finally, we reviewed 13 articles that included 14 human cases (Fig. 3, Table 2).

**Table 2:** Summary of the reported human cases with empyema necessitans associated with *MRSA*

Case	Reference	Age	Sex	Year	Underlying diseases	Isolate lesion	Disseminated lesion	Invasive procedures	Treatment	Outcome
1	Kugai et al. [7]	49 years	Male	1993	LC, immediately after direct surgical intervention for Budd–Chiari syndrome, left lower leg ulcer caused by MRSA 3 months previously	Sputum, pleural fluid	None	Open pleural drainage, continuous intrathoracic irrigation (Povidone Iodine Solution 1% 100 mL + saline 500 mL)	flomoxef 7 days → imipnem/cilastatin + NET 2 days → MIN + NET 3 days → MIN + ABK 4 days → VAN 4 days → + OFX 16 days → FOF + OFX 20 days → VAN + OFX 8 days → flomoxef + OFX 6 days → OFX 10 days	Complete resolution
2	Stallworth et al. [3]	8 months	Male	2005	None	Blood	None	Thoracotomy	AZM → CTX → + VAN 10 days → SXT (PO) to complete a 21-day course of antibiotics	Complete resolution
3	Moore et al. [8]	3 months	Female	2006	None	Intraoperative cultures from the right chest wall	None	Decortication, tube thoracostomy, wide drainage of the subscapular collection	CRO + VAN to complete a total of 14 days → LZD 7 days	Complete resolution
4	Mizell et al. [5]	59 years	Male	2008	HT, DM, CRF, LC	Blood, urine, aspiration fluid from chest mass	Osteomyelitis of the left lateral portion of the sternum and the distal end of the first rib	Fine-needle aspiration of the chest wall mass, wedge resection of the left upper lung lobe with tube thoracostomy drainage	VAN 25 days → CIP + SXT (PO) 10 days	Not reported
5	Contreras et al. [9]	19 months	Male	2009	None	Blood, chest wall fluid	Septic emboli, acute osteomyelitis of the right distal femur	Thoracoscopic decortication	AZM → CRO + VAN → GEN + VAN 2 weeks → VAN 36 days → CLI (PO)	Complete resolution
6	Rosebush et al. [10]	4 weeks	Female	2014	Exposure to a maternal breast abscess caused by MRSA via breast-feeding	Fluid from intervention	Osteomyelitis of the posterolateral right 9th, 10th, and 11th ribs	Tube thoracostomy (pigtail catheter)	AMP + GEN + CLI → AMP + CLI + CTX → CLI (IV) to complete a total of 4 weeks → CLI (PO) 4 weeks	Complete resolution
7	Lee et al. [11]	1 year and 8 months	Female	2015	None	Fluid from intervention	None	Thoracoscopic decortication, tube thoracotomy	AMC → LZD 21 days	Not reported
8	Edriss et al. [12]	60 years	Male	2017	Tobacco abuse, remote alcohol abuse, and left total hip arthroplasty presented	Sputum	None	Wedge resection of the left upper lobe	VAN + MEM to complete a total of 6–8 weeks	Complete resolution
9	Pugh et al. [13]	5 years	Male	2019	Obstructive hydrocephalus status postventriculoatrial shunt placement, influenza A infection 1 week before admission	Pleural fluid	None	Removal of the shunt system, tube thoracotomy, infused with fibrinolytic agent alteplase	FEP + VAN → + CLI → VAN to complete a total of 21 days	Complete resolution
10	Pankaj et al. [14]	63 years	Male	2021	Not reported	Blood	None	Tube thoracotomy	VAN + CRO + AZM to complete a total of 14 days	Not reported
11	Farouji et al. [15]	35 years	Female	2021	Active intravenous drug use	Fluid from intervention	None	Interventional radiology-guided incision and drainage → a left upper lobectomy	TZP + VAN → VAN to complete a total of 4 weeks	Not reported
12	Ashraf et al. [16]	2 months	Female	2022	None	Pus from intervention/4th rib osteomyelitis	Left 4th rib osteomyelitis	Tube thoracotomy, incision and drainage of the abscess	CRO + AMC → TZP + VAN for 3 weeks	Complete resolution
13	Ashraf et al. [16]	10 years	Male	2022	History of trauma to the right knee 7 days previously	Pus from the right knee and pleural fluid	Right knee, anterior aspect of the right upper arm, quadratus lumborum muscle, bilateral gluteal muscles and obturator interni	Tube thoracotomy, surgical drainage of right knee abscess, incision on upper back	CRO + VAN → sulbactam/cefoperazone to complete a total of 6 weeks	Complete resolution
14	Rehman et al. [17]	1.5 years	Female	2023	None	Blood, pleural fluid	None	Tube thoracotomy and decortication (two chest tubes placed)	CRO + VAN 3 days → LZD (IV) 4 days → LZD (PO) for 4 weeks	Complete resolution
Our case	Nakamura et al	69 years	Male	2023	Stones in the urinary tract	Blood	Cervical osteomyelitis, paravertebral inflammation	Left thoracotomy	SAM → + VAN → + DAP 4 weeks → LZD 4 weeks, VAN 14 days → SXT (PO) 44 days → DAP 3 days → MIN	Relapse → Complete resolution

*Abbreviations*: *LC* liver cirrhosis, *NET* netilmicin, *MIN* minocycline, *MRSA* methicillin-resistant *Staphylococcus aureus*, *ABK* arbekacin, *OFX* ofloxacin, *FOF* fosfomycin, *VAN* vancomycin, *AZM* azithromycin, *CTX* cefotaxime, *SXT* trimethoprim–sulfamethoxazole, *PO* oral administration, *CRO* ceftriaxone, *LZD* linezolid, *HT* hypertension, *DM* diabetes mellitus, *CRF* chronic renal failure, *CIP* ciprofloxacin, *GEN* gentamicin, *CLI* clindamycin, *AMP* ampicillin, *AMC* amoxicillin–clavulanic acid, *MEM* meropenem, *FEP* cefepime, *TZP* piperacillin/tazobactam, *SAM* ampicillin/sulbactam, *DAP* daptomycin

## Discussion and conclusion

EN is a rare clinical condition that was first described by Gullan De Baillon in 1640; it refers to an infection of the pleura and the associated spread of pus beyond the pleural cavity into adjacent soft tissue structures [5]. EN typically results from necrotizing pneumonia that has been present for a long period and may also occur after other trauma or open chest surgery [18]. *M. tuberculosis* and *A. israelii* are usually identified from pleural effusions as the main causative agents [2–4]. However, EN caused by MRSA is rare. Although there are reports of an overall mortality rate of 66% in the pre-antimicrobial era of EN [1], limited information is available [6]. Based on the above, EN caused by MRSA has rarely been reported, but is expected to be more severe. We reported the case of EN caused by MRSA, and owing to its rarity, we performed a literature review to investigate its complications, management, and prognosis.

Fifteen patients, including the present patient, were included in the literature review. The patients consisted of six adults (median age: 59 years, range: 35–69 years) and nine children (median age, 1.5 years, range: 4 weeks–10 years), with a slightly larger proportion of males (*n* = 10, 67%). The proportions of underlying diseases and risk factors were lower in children (*n* = 3, 33%) and higher in adults (*n* = 5, 83%). Factors such as liver cirrhosis (*n* = 2), postoperative state (*n* = 2), and diabetes mellitus (*n* = 1) were identified in the adult patients. Blood culture positivity was present in 40% (*n* = 8) and disseminated lesions were present in 30% (*n* = 6) of the patients. Disseminated lesions included three cases of osteomyelitis of the ribs due to direct deep penetration of the empyema, two cases of osteomyelitis as metastatic lesions, and one case each of multiple intramuscular abscesses and septic pulmonary embolism. MRSA infections cause metastatic infections [19]. It is particularly important to identify metastatic infections when blood cultures are positive, as in the present case [20]. Osteomyelitis, especially in our literature review, is frequently reported and should be considered for diagnosis using MRI if suspected.

Regarding surgical interventions, 73% (*n* = 11) had a tube thoracostomy, 33% (*n* = 5) an approach to a subcutaneous abscess, 27% (*n* = 4) thoracoscopic decortication, 20% (*n* = 3) a partial lung resection, and one patient each had used an intrapleural fibrinolytic agent, open chest surgery, continuous intrathoracic irrigation, and removal of the prosthesis. All patients underwent some form of surgical intervention, and none received antimicrobial therapy alone. Regarding the prognosis, no death cases were reported. A high mortality rate for MRSA empyema has been described in previous reports; however, there is no description of its actual treatment [6]. In all cases in this review, appropriate surgical intervention was performed, which may have led to a better prognosis. We should consider the aggressive drainage with reference to the treatment performed in this literature review. Furthermore, there has been only one case of treatment failure in the literature review, which was our case. In our case, the disseminated lesion was vertebral osteomyelitis, and MRSA osteomyelitis has a high relapse rate, with approximately 30% of patients relapsing after less than eight weeks of treatment [21]. Therefore, anti-MRSA agents were initially administered in this case for eight weeks. However, in retrospect, the patient had a high ESR of 80 mm/h at eight weeks and may have been at high risk of recurrence. Even with the appropriate treatment for empyema, some metastatic infection sites can be difficult to treat, and clinicians should pay close attention when treating metastatic infection.

In this case, daptomycin was added after confirming persistent bacteremia during the VAN continuation. Therapeutic drug monitoring (TDM) with VAN is recommended with a ratio of area under the curve over 24 h to minimum inhibitory concentration (AUC/MIC) of ≥ 400 [22]. Since trough concentrations in the range of 15–20 μg/mL may be compatible with an AUC/MIC of ≥ 400 [23], we implemented trough-guided TDM according to the available resources at our hospital. VAN is widely prescribed and the first choice of treatment for MRSA bacteremia [24]. However, several disadvantages associated with VAN administration have been reported, including low tissue penetration, slow bactericidal effect, and the emergence of resistant strains during treatment [25]. In our case, although we maintained an optimal trough for VAN, we were unable to achieve a negative conversion of MRSA bacteremia. In recent years, several reports have demonstrated a significant reduction in 30-day mortality when daptomycin was initiated within 72 h of the onset of MRSA bacteremia [26–28]. In addition, daptomycin is associated with good tissue transfer, and the successful treatment of empyema by switching from linezolid to VAN has been reported in some cases [29, 30]. According to some reports, daptomycin is less likely than VAN to cause clinical failure [31]. In the case of MRSA, which has a high mortality rate, studies are being conducted using a combination of anti-MRSA drugs and the beta-lactam antibiotics SXT and fosfomycin; however, good results in terms of patient outcomes have not been obtained. With the combination of daptomycin and VAN, some older studies have demonstrated that all *Staphylococcus aureus* isolates develop daptomycin nonsusceptibility in the presence of VAN [32]. Based on the results of this study, there have been few studies on combined therapy with daptomycin and VAN. However, there is a lack of clear evidence on the deterioration associated with combined therapy [33, 34]. Daptomycin cannot be used for microorganisms via the alveoli because it is inactivated by a type 2 surfactant; however, it can be used effectively for empyema. Therefore, we believe that a combination of VAN and daptomycin can be used effectively and safely to treat empyema.

In EN as a whole, several cases were reported in the preantibiotic era, and in recent years, the number of cases has been declining, regardless of the bacterial species [35]. It has been suggested that this is due to the fact that in most cases of infection, patients respond quickly to antimicrobial agents when properly diagnosed [36]. Therefore, the diagnosis of EN in the modern era may be the result of delayed diagnosis or severe cases. A review of the period 1966–2004, when antimicrobial agents were widely used, revealed that *M. tuberculosis* and *A. israelii* were the most common causative organisms [1]. In contrast, a 2010 review noted an increase in the frequency of MRSA as a cause [36]. In our review, 66% (10/15) of the cases occurred from 2010 onward. In summary, even the usual MRSA empyema is difficult to treat in the first place, while MRSA EN is likely to be even more severe. Recently, the severity of EN as a disease has increased. The importance of MRSA EN as a disease has increased as well in recent years.

As discussed above, compared to usual empyema, the causative pathogens of EN are more frequently represented by MRSA or tuberculosis, even in the community-acquired infections. These causative pathogens cannot be eradicated by empirical therapy alone, which is commonly used to treat empyema [2–4, 37]. Therefore, identifying the causative organism is more important in the EN than in usual empyema. Drainage should be performed first when EN is suspected, both for therapeutic purposes and to identify the causative organism. In the present case, drainage was performed on the day the patient arrived for prompt diagnosis and appropriate treatment.

In conclusion, we encountered a rare case of empyema caused by MRSA. Early drainage of the empyema should be performed to identify the causative pathogen and develop an optimal management strategy.

### Supplementary Information

**Additional file 1. **Search terms used to search three databases (PubMed, Embase, and Ichushi) for literature reviews on empyema necessitans associated with MRSA.

## Data Availability

Not applicable.

## Abbreviations

AUC: Area Under the Curve
CT: Computed Tomography
EN: Empyema Necessitans
ESR: Erythrocyte Sedimentation Rate
LDH: Lactate Dehydrogenase
MIC: Minimum Inhibitory Concentration
MRI: Magnetic Resonance Imaging
MRSA: Methicillin-Resistant *Staphylococcus aureus*
SXT: Sulfamethoxazole–Trimethoprim
TDM: Therapeutic Drug Monitoring
VAN: Vancomycin
