# Supplementary material for: Empyema necessitans caused by methicillin-resistant Staphylococcus aureus: a case report and literature review
Source: BMC Infect Dis. 2024 Feb 1;24:157. doi: 10.1186/s12879-024-09062-0 (PMC10835956; doi:10.1186/s12879-024-09062-0)
Supplement: Supplementary file 1 — Additional file 1. Search terms used to search three databases (PubMed, Embase, and Ichushi) for literature reviews on empyema necessitans associated with MRSA. [file 12879_2024_9062_MOESM1_ESM.docx]

Additional file 1. Search terms used to search three databases (PubMed, Embase, and Ichushi) for literature reviews on empyema necessitans associated with MRSA.

| PubMed | ("Methicillin-Resistant Staphylococcus aureus"[MeSH Terms] OR "MRSA"[Title/Abstract] OR "EMRSA"[Title/Abstract] OR (("Penicillin Resistance"[MeSH Terms] OR (("methicillin"[Title/Abstract] OR "methillicin"[Title/Abstract] OR "meticillin"[Title/Abstract] OR "penicillin"[Title/Abstract]) AND "resist*"[Title/Abstract]) OR "drug resistance microbial"[Text Word]) AND ("Staphylococcus aureus"[MeSH Terms] OR "Staphylococcal Infections"[MeSH Terms] OR "Staphylococcus aureus"[Title/Abstract:~3] OR "Staphylo coccus aureus"[Title/Abstract:~3] OR "staphylococcusaureus"[Title/Abstract] OR "Staphylococcal Infection"[Title/Abstract:~3] OR "Staphylococcal Infections"[Title/Abstract:~3] OR "staphylo coccal infection"[Title/Abstract:~3] OR "staphylo coccal infections"[Title/Abstract:~3]))) AND ("empyema"[MeSH Terms] OR "empyema*"[Title/Abstract] OR "empyema, pleural"[MeSH Terms] OR "Pyothorax"[Title/Abstract] OR (("pleural"[Title/Abstract] OR "pleurisy"[Title/Abstract] OR "pleuri*"[Title/Abstract] OR "parapneumonic"[Title/Abstract]) AND ("suppurati*"[Title/Abstract] OR "purulent"[Title/Abstract] OR "effusion*"[Title/Abstract]))) |
| --- | --- |
| Embase | ('empyema'/exp OR 'pleura empyema'/exp OR empyema*:ti,ab,kw OR pyothorax:ti,ab,kw OR (((pleural OR pleurisy OR pleuri* OR parapneumonic) NEAR/3 (suppurati* OR purulent OR effusion*)):ti,ab,kw)) AND ('methicillin resistant staphylococcus aureus'/exp OR mrsa:ti,ab,kw OR emrsa:ti,ab,kw OR (('methicillin resistance'/exp OR (((methicillin OR methillicin OR meticillin OR penicillin) NEAR/3 resist*):ti,ab,kw) OR (('drug resistan*' NEAR/3 microbial):ti,ab,kw)) AND ('staphylococcus aureus'/exp OR ((staphylococcus NEAR/3 aureus):ti,ab,kw) OR (('staphylo coccus' NEAR/3 aureus):ti,ab,kw) OR 'staphylococcusaureus':ti,ab,kw OR ((staphylococcus NEAR/3 infection*):ti,ab,kw) OR 'staphylococcus infection'/exp))) |
| Ichushi | ((((Metchcillin-resistant staphylcoccus aurues[Japanese]/TH) or (MRSA/TA) or (EMRSA/TA)) or (((penicillin-resistant[Japanese]/TH) or (Metchcillin[Japanese]/TA and resistant[Japanese]/TA) or (Methicillin/TA and resistant[Japanese]/TA)) and (("Staphylococcus aureus"/TH) or (staphylococcus infection[Japanese]/TH) or (staphylococcus aureus[Japanese]/TA) or (staphylococcus[Japanese]/TA) or (staphylocccus-like[Japanese]/TA) or (staphylococcus[[Japanese]]/TA) or (staphyloccocus-like[Japanese]/TA)))) and (((emypema[Japanese]/TH or empyema[Japanese]AL)) or (pyogenic pleuritis/TA) or (pyogenic pleural effusion[Japanese]/TA) or ((chestwall[Japanese]/TA or pleural cavity[Japanese]/TA or pleural cavity[Japanese]/TA) and (abscess[Japanese]/TA or @"abscess[Japanese]"/TH)))) |
